# Dietary supplementary glutamine and L-carnitine enhanced the anti-cold stress of Arbor Acres broilers

**DOI:** 10.5194/aab-64-231-2021

**Published:** 2021-06-04

**Authors:** Yang Liu, Yuying Yang, Ruizhi Yao, Yajie Hu, Peng Liu, Shuai Lian, Hongming Lv, Bin Xu, Shize Li

**Affiliations:** 1 National Experimental Teaching Demonstration Center of Animal Medicine Foundation, College of Animal Science and Veterinary Medicine, Heilongjiang Bayi Agricultural University, Daqing, 163319, PR China; 2 College of Animal Science and Technology, Inner Mongolia University for Nationalities, Tongliao, 028000, PR China

## Abstract

Newborn poultry in cold regions often suffer from cold stress, causing a series of changes in their physiology and metabolism, leading to slow growth and decreased production performance. However, a single anti-stress substance cannot completely or maximally eliminate or alleviate the various effects of cold stress on animals. Therefore, the effects of the supplemented glutamine and L-carnitine on broilers under low temperature were evaluated in this study. Broilers were randomly allocated into 16 groups which were respectively fed with different levels of glutamine and L-carnitine according to the L${}_{16}$ (4${}^{5}$) orthogonal experimental design for 3 weeks (the first week is the adaptive feeding period; the second and third weeks are the cold exposure period). Growth performance was recorded, and blood samples were collected during cold exposure. The results showed the supplementation had altered the plasma parameters, growth performance and cold-induced oxidative stress. The increase of corticosterone and suppression of thyroid hormone was ameliorated. Supplemented groups had lower daily feed intake and feed-to-gain ratio, higher daily weight gain and better relative weights of immune organs. Plasma glucose, total protein, blood urea nitrogen and alkaline phosphatase changed as well. Oxidative stress was mollified due to the improved activities of superoxide dismutase and glutathione peroxidase, heightened total antioxidant capacity and stable malondialdehyde. Dietary glutamine and L-carnitine improve the growth performance, nutritional status and cold stress response of broilers at low temperature, and their interaction occurred.

## 1 Introduction

Within the background of global warming, the environment becomes more complex and changeable, and the disturbance to animals becomes more frequent (Bailey et al., 2019; Hansen et al., 2019). At present, environmental low temperature has become one of the most common stressors in cold regions, and the cold stress caused by it poses a threat to the health of human beings and animals. Cold stress is able to induce various negative responses like other stresses, affecting neuroendocrine, reproduction and cardiovascular systems, to change the biochemical catalytic processes of the organism, leading to oxidative damage, apoptosis, and other physiological or pathological reactions (García-Díaz et al., 2015; Solianik et al., 2015; Lian et al., 2017; Zhang et al., 2017; Cong et al., 2018). Cold stress also significantly increases systemic energy expenditure and causes substantial metabolic changes, including decreased insulinemia, increased hepatic glucose production, feed intake changes, and increased glucose and fat utilization in peripheral tissues (Yao et al., 2018; Liu et al., 2019; Shi et al., 2019). Meanwhile, cold stress affects the production performance and product quality of livestock and poultry (Zhang et al., 2014; Hao et al., 2015; Nguyen et al., 2015). For example, cold stress not only stunts the growth and development of broilers, but also requires more feed to maintain their body temperature, resulting in low feed conversion rate and waste of resources, as well as poor weight uniformity and carcass quality of broilers. More seriously, the morbidity and mortality will be exacerbated (Tsiouris et al., 2015). Therefore, cold stress is one of the main barriers limiting the development of animal husbandry and is an urgent problem to be solved in cold regions.

At present, one of the most effective ways to resist cold stress is to improve diet. However, stress response is a non-specific systemic adaptive response and its physiological abnormalities are also various. Therefore, single anti-stress additives or nutritional supplements are difficult to promote the maintenance of body homeostasis.

Finally, we selected the combination of glutamine and L-carnitine to increase the nutritional and energy levels of the diet to improve the adverse reactions of cold stress. Glutamine is the most abundant free amino acid of the body (Wellen et al., 2010; Cruzat et al., 2018; Li et al., 2019). Besides its role as a constituent of proteins and its importance in amino acid transamination, glutamine has many kinds of nonnutritive function (Li et al., 2019) and has been claimed to be essential conditionally during stress or clinical situations (Stehle and Kuhn, 2015; Coqueiro et al., 2019; Oh et al., 2020). For example, glutamine is the principal metabolic fuel for small intestine enterocytes, lymphocytes, macrophages and fibroblasts and is considered an essential amino acid in some species under inflammatory conditions such as infection and injury (Roth, 2008; Carr et al., 2010). Moreover, glutamine is one of the most efficacious substrates for gluconeogenesis and has a protective effect on the physiology and function of gastrointestinal tract (Fan et al., 2015; Zuhl et al., 2015). Glutamine is able to decrease the incidence of infection in surgery and trauma patients and is investigated as one effective supplementary nutrition (Moe-Byrne et al., 2012). Meanwhile, L-carnitine is reported to have two major functions: facilitating the transport of long-chain fatty acids across the mitochondrial membrane and the removal from mitochondria of short- and medium-chain fatty acids that accumulate as a result of normal and abnormal metabolism (Rehman et al., 2017; Weinert et al., 2017). Thus, dietary L-carnitine supplementation promotes the β-oxidation of these fatty acids to generate adenosine triphosphate (ATP) and improves energy utilization (Madsen et al., 2018). In addition, L-carnitine participates in biological processes, for example, regulation of gluconeogenesis, stimulation of fatty acid synthesis and ketone, branched-chain amino acid, triglyceride, and cholesterol metabolism (Maruyama et al., 2019). L-carnitine therapy is also a reasonable approach for reducing systemic inflammation and its complications (Khalatbari-Soltani and Tabibi, 2015).

The dietary addition of glutamine and L-carnitine has been studied a lot. But the research about the glutamine supplementation is mainly focused on its oral, parenteral, or enteral application under clinical conditions (Roth, 2008; Tian et al., 2017). A similar state also exists in the research field of L-carnitine (Mongioi et al., 2016; Agarwal et al., 2018; Gavrilova, 2018). However, there are few reports about the effects of dietary glutamine and L-carnitine on anti-cold stress of broilers. Therefore, the purpose of this study is to investigate the influence of the supplemental glutamine and L-carnitine on the grow performance, the physiological response and antioxidant capacity of Arbor Acres (AA) broiler chickens under cold exposure.

## 2 Materials and methods

### 2.1 Chickens and diets

**Figure 1:**
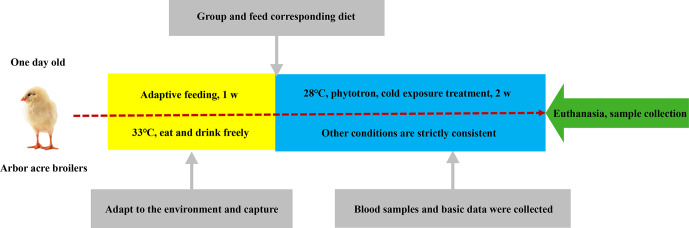
Timeline of the treatment protocol.

In this study, 480 one-day-old Arbor Acres (AA) broiler chickens were randomly divided into 16 treatment groups of 30 birds each. Each group was further divided into one main and two replicate subgroups. Each group was kept in a specially designed biotron (College of Animal Science and Veterinary Medicine, Heilongjiang Bayi Agricultural University), where the temperature, relative humidity and lighting were controlled. These 16 groups were administrated according to the L${}_{16}$ (4${}^{5}$) orthogonal experimental design of two diet supplements: glutamine (0, 0.4, 0.6, 0.8 percentage of diet weight) and L-carnitine (0, 50, 75, 100 mg/kg diet weight). The levels of glutamine and L-carnitine in the diet are shown in Table 1. The basal meal met the National Research Council requirements. The experimental diets and drinking water were offered ad libitum. After the first week of adaptive feeding, all groups were exposed to cold temperatures at 23±0.5 ${}^{\circ }$C (this ambient temperature went down by 10±0.5 ${}^{\circ }$C compared to the optimal temperature of 33 ${}^{\circ }$C, and the relative humidity was 40 %) for 2 weeks. Feed intake and body weight gain were recorded weekly during the 2-week cold exposure period, and daily feed intake (DFI), daily weight gain (DWG) and feed-to-gain ratio (F / G) were calculated. The experimental scheme is shown in Fig. 1.

**Table 1:** Orthogonal experimental design L${}_{16}$ (4${}^{5}$).

Group	Glutamine	L-carnitine
	(%)	(mg/kg)
A${}_{1}$B${}_{1}$	0.4	50
A${}_{1}$B${}_{2}$	0.4	75
A${}_{1}$B${}_{3}$	0.4	100
A${}_{1}$B${}_{4}$	0.4	0.0
A${}_{2}$B${}_{1}$	0.6	50
A${}_{2}$B${}_{2}$	0.6	75
A${}_{2}$B${}_{3}$	0.6	100
A${}_{2}$B${}_{4}$	0.6	0.0
A${}_{3}$B${}_{1}$	0.8	50
A${}_{3}$B${}_{2}$	0.8	75
A${}_{3}$B${}_{3}$	0.8	100
A${}_{3}$B${}_{4}$	0.8	0.0
A${}_{4}$B${}_{1}$	0.0	50
A${}_{4}$B${}_{2}$	0.0	75
A${}_{4}$B${}_{3}$	0.0	100
A${}_{4}$B${}_{4}$	0.0	0.0

Blood samples were collected weekly from each group at 06:00 GMT+8 of each blood collection day. Blood was drawn from a wing vein using a heparinized syringe within 30 s, applied to the warm bath at 37 ${}^{\circ }$C for 20 min and then centrifuged at 3500 rpm for 10 min to obtain plasma, which was stored at -70 ${}^{\circ }$C until analysis. All groups were euthanized after 12 h of feed deprivation at the end of cold exposure. The weights of spleen, thymus and bursa of Fabricius were determined, and relative weights of each organ were calculated.

All experiments were approved by animal protection and the Utilization Committee of Heilongjiang Bayi Agricultural University. All operations were carried out according to the regulations of animal science and veterinary medicine college. All efforts were made to minimize animal suffering and to reduce the number of animals used.

### 2.2 Measurement of biochemical parameters

Corticosterone (CORT), as a classical stress hormone, was selected to evaluate the cold stress state of chickens in each group. Thyroid hormones (triiodothyronine, T${}_{3}$ and thyroxine, T${}_{4}$) have been selected to assess the functional status of the thyroid body, which is related to the body's metabolic heat production and body heat regulation. Plasma CORT, T${}_{3}$ and T${}_{4}$ were measured using a commercial kit (Nanjing Jiancheng Bioengineering Institute, Nanjing, PR China).

Plasma glucose (GLU), total protein (TP), blood urea nitrogen (BUN) and alkaline phosphatase (ALP) have been used as indicators to evaluate the biochemical and metabolic changes of chicks in each group and were measured using a commercial kit (Nanjing Jiancheng Bioengineering Institute, Nanjing, PR China).

Plasma superoxide dismutase (SOD), glutathione peroxidase (GSH-PX), total antioxidant capacity (T-AOC) and malondialdehyde (MDA) were used to assess the antioxidant capacity of chicks in each group and were measured using a commercial kit (Nanjing Jiancheng Bioengineering Institute, Nanjing, PR China).

All operations were carried out according to the instructions of the kit.

### 2.3 Statistical analysis

The effects of glutamine and L-carnitine on the measured parameters and the possible time-dependent effects were analyzed by the general linear model (SPSS Ver. 19.0). Duncan's test was used to determine which specific pairs differed. Means were considered significantly different when P<0.05.

## 3 Results

### 3.1 Effects of glutamine and L-carnitine on growth performance and immune organ index of broilers under cold stress

The DFI, DWG and F / G are presented in Table 2, the relative weights of the spleen, thymus and bursa of Fabricius in Table 3, and the results of orthogonal contrast and variance analysis in Table 4. The growth performance of the broilers exposed to the low ambient temperature improved due to the supplementation of glutamine and L-carnitine. In the first week, the added glutamine and L-carnitine had obviously altered the DFI, DWG and F / G (P<0.05), whereas there was an interaction only on DWG and F / G (P<0.05). Groups A${}_{1}$ or B${}_{1}$ had the highest DFI, but the lowest DWG and the highest F / G. On the contrary, in the second week, except for DFI neither DWG nor F / G was affected by the two additives and their interaction (P>0.05). Groups A${}_{1}$ or B${}_{1}$ still had the biggest DFI means and relatively lower DWG and F / G but did not maintain the least levels. With regard to the total values, the changes of DFI, DWG and F / G induced by the supplementation were significant (P<0.05), and there was apparent interaction (P<0.05). Groups A${}_{1}$ or B${}_{1}$ had the high DFI, the lowest DWG and the highest F / G. Overall, the DFI and F / G of the broilers in group A${}_{1}$B${}_{1}$ were the highest while the DWG was not the least.

**Table 2:** The growth performance of Arbor Acres broilers under low temperature conditions supplemented by glutamine and L-carnitine.

Group	Daily feed intake (g/d)			Daily weight gain (g/d)			Feed-to-gain ratio (F / G)		
	2 week	3 week	total	2 week	3 week	total	2 week	3 week	total
A${}_{1}$B${}_{1}$	52.39 ± 1.03	74.48 ± 8.67	65.38 ± 3.35	32.16 ± 1.91	36.20 ± 1.42	34.52 ± 1.50	1.72 ± 0.09	2.18 ± 0.23	1.95 ± 0.08
A${}_{1}$B${}_{2}$	48.53 ± 2.60	71.12 ± 6.62	58.21 ± 3.87	32.20 ± 2.15	37.94 ± 1.58	35.57 ± 3.45	1.41 ± 0.03	2.01 ± 0.37	1.71 ± 0.11
A${}_{1}$B${}_{3}$	50.29 ± 3.02	72.36 ± 0.46	62.38 ± 3.72	34.64 ± 1.92	44.84 ± 2.48	38.74 ± 2.94	1.46 ± 0.16	1.95 ± 0.67	1.68 ± 0.30
A${}_{1}$B${}_{4}$	45.62 ± 2.63	73.06 ± 2.74	61.08 ± 2.40	32.85 ± 3.45	43.47 ± 3.86	36.33 ± 1.19	1.71 ± 0.23	1.95 ± 0.08	1.74 ± 0.17
A${}_{2}$B${}_{1}$	45.30 ± 3.42	70.69 ± 3.45	59.62 ± 3.02	33.85 ± 1.58	35.79 ± 1.13	34.82 ± 2.16	1.43 ± 0.12	1.89 ± 0.31	1.72 ± 0.19
A${}_{2}$B${}_{2}$	47.01 ± 2.97	69.80 ± 2.33	58.41 ± 2.65	31.75 ± 2.68	44.02 ± 3.72	37.88 ± 3.53	1.48 ± 0.08	1.62 ± 0.28	1.58 ± 0.06
A${}_{2}$B${}_{3}$	45.10 ± 2.01	69.38 ± 0.62	57.24 ± 2.41	32.50 ± 2.94	41.01 ± 2.45	36.76 ± 5.23	1.37 ± 0.15	1.80 ± 0.59	1.59 ± 0.27
A${}_{2}$B${}_{4}$	46.90 ± 2.17	71.75 ± 0.71	59.32 ± 0.74	33.89 ± 3.74	45.15 ± 2.92	38.02 ± 3.31	1.39 ± 0.16	1.74 ± 0.10	1.56 ± 0.13
A${}_{3}$B${}_{1}$	46.90 ± 1.45	72.07 ± 1.24	59.49 ± 1.74	34.37 ± 1.27	41.80 ± 2.74	38.09 ± 2.86	1.47 ± 0.04	1.75 ± 0.24	1.65 ± 0.11
A${}_{3}$B${}_{2}$	48.39 ± 3.12	65.14 ± 6.09	56.77 ± 3.95	34.68 ± 4.02	40.97 ± 1.75	37.82 ± 2.38	1.55 ± 0.29	1.59 ± 0.10	1.57 ± 0.17
A${}_{3}$B${}_{3}$	49.10 ± 2.62	67.19 ± 1.07	58.26 ± 2.36	36.90 ± 1.52	47.58 ± 2.31	40.23 ± 4.70	1.36 ± 0.16	1.71 ± 0.31	1.55 ± 0.14
A${}_{3}$B${}_{4}$	44.48 ± 4.29	67.22 ± 3.81	57.57 ± 1.70	33.89 ± 4.91	40.31 ± 1.69	35.89 ± 0.61	1.42 ± 0.33	1.57 ± 0.11	1.49 ± 0.12
A${}_{4}$B${}_{1}$	48.42 ± 1.84	72.74 ± 4.25	60.59 ± 2.93	34.70 ± 1.74	40.60 ± 1.05	37.65 ± 2.18	1.51 ± 0.07	1.81 ± 0.23	1.60 ± 0.10
A${}_{4}$B${}_{2}$	49.90 ± 1.98	71.20 ± 3.75	59.99 ± 0.91	34.18 ± 1.04	37.48 ± 2.10	35.83 ± 3.29	1.46 ± 0.25	1.79 ± 0.49	1.63 ± 0.22
A${}_{4}$B${}_{3}$	51.86 ± 1.63	68.11 ± 2.47	56.41 ± 1.81	33.49 ± 1.87	39.89 ± 2.27	37.73 ± 1.71	1.39 ± 0.10	1.95 ± 0.16	1.73 ± 0.08
A${}_{4}$B${}_{4}$	47.91 ± 4.63	64.11 ± 6.74	57.31 ± 3.41	33.52 ± 3.45	45.38 ± 1.26	36.90 ± 6.78	1.41 ± 0.10	1.87 ± 0.08	1.57 ± 0.07

The groups A${}_{1}$, A${}_{2}$, A${}_{3}$ and A${}_{4}$ were treated by glutamine at 0, 0.4, 0.6 and 0.8 percentage of diet weight, and groups B${}_{1}$, B${}_{2}$, B${}_{3}$ and B${}_{4}$ were treated by L-carnitine at 0, 50, 75 and 100 mg/kg diet weight. Data are given as mean ± SEM.

**Table 3:** The immune organ indexes of Arbor Acres broilers under low temperature conditions supplemented by glutamine and L-carnitine.

Group	Relative	Relative	Relative
	weight of	weight of	weight of
	spleen	thymus	bursa of
	(mg/g)	(mg/g)	Fabricius
			(mg/g)
A${}_{1}$B${}_{1}$	0.64 ± 0.05	2.03 ± 0.08	2.00 ± 0.27
A${}_{1}$B${}_{2}$	0.75 ± 0.07	2.17 ± 0.07	2.30 ± 0.10
A${}_{1}$B${}_{3}$	0.82 ± 0.04	2.35 ± 0.06	2.04 ± 0.07
A${}_{1}$B${}_{4}$	0.67 ± 0.15	2.23 ± 0.08	2.35 ± 0.36
A${}_{2}$B${}_{1}$	0.70 ± 0.06	2.41 ± 0.30	2.27 ± 0.08
A${}_{2}$B${}_{2}$	0.78 ± 0.12	2.56 ± 0.73	2.44 ± 0.26
A${}_{2}$B${}_{3}$	0.74 ± 0.12	2.86 ± 0.18	2.39 ± 0.17
A${}_{2}$B${}_{4}$	0.74 ± 0.05	2.91 ± 0.54	2.56 ± 0.49
A${}_{3}$B${}_{1}$	0.74 ± 0.03	2.28 ± 0.26	2.37 ± 0.07
A${}_{3}$B${}_{2}$	0.83 ± 0.04	2.51 ± 0.15	2.41 ± 0.46
A${}_{3}$B${}_{3}$	0.98 ± 0.11	3.55 ± 0.12	2.79 ± 0.16
A${}_{3}$B${}_{4}$	1.02 ± 0.05	2.96 ± 0.43	2.47 ± 0.13
A${}_{4}$B${}_{1}$	0.83 ± 0.09	2.47 ± 0.68	2.24 ± 0.21
A${}_{4}$B${}_{2}$	0.86 ± 0.04	2.63 ± 0.25	2.23 ± 0.11
A${}_{4}$B${}_{3}$	0.93 ± 0.09	2.67 ± 0.39	2.39 ± 0.19
A${}_{4}$B${}_{4}$	0.76 ± 0.10	3.12 ± 0.15	2.61 ± 0.12

The groups A${}_{1}$, A${}_{2}$, A${}_{3}$ and A${}_{4}$ were treated by glutamine at 0, 0.4, 0.6 and 0.8 percentage of diet weight, and groups B${}_{1}$, B${}_{2}$, B${}_{3}$ and B${}_{4}$ were treated by L-carnitine at 0, 50, 75 and 100 mg/kg diet weight. Data are given as mean ± SEM.

**Table 4:** The effects of supplemented glutamine and L-carnitine on growth performance and organ's relative weight of Arbor Acres broilers under low temperature conditions.

Group	Daily feed intake (g/d)			Daily weight gain (g/d)			Feed-to-gain ratio (F / G)			Relative	Relative	Relative
	2	3	total	2	3	total	2	3	total	weight of	weight of	weight of
	week	week		week	week		week	week		spleen	thymus	bursa of
												Fabricius
Glutamine												
A${}_{1}$	49.27${}^{a}$	72.06${}^{a}$	58.82	32.61${}^{b}$	40.37	36.03${}^{b}$	1.50	1.86	1.65	0.71${}^{b}$	2.54	2.27${}^{b}$
A${}_{2}$	48.10${}^{\mathrm{ab}}$	71.05${}^{\mathrm{ab}}$	60.08	32.96${}^{\mathrm{ab}}$	41.49	36.87${}^{\mathrm{ab}}$	1.49	1.85	1.63	0.80${}^{b}$	2.49	2.42${}^{\mathrm{ab}}$
A${}_{3}$	46.58${}^{b}$	69.38${}^{\mathrm{ab}}$	59.30	32.25${}^{\mathrm{ab}}$	42.87	36.16${}^{\mathrm{ab}}$	1.41	1.68	1.6	0.95${}^{a}$	2.52	2.47${}^{a}$
A${}_{4}$	48.08${}^{\mathrm{ab}}$	69.33${}^{b}$	58.73	34.19${}^{a}$	44.16	37.89${}^{a}$	1.44	1.79	1.58	0.79${}^{b}$	2.58	2.31${}^{\mathrm{ab}}$
L-carnitine												
B${}_{1}$	48.93	73.26	60.10	32.01${}^{b}$	42.85	35.65${}^{b}$	1.52	1.80	1.68	0.77${}^{c}$	2.29${}^{b}$	2.37
B${}_{2}$	47.65	70.17	59.49	32.45${}^{\mathrm{ab}}$	42.29	37.03${}^{\mathrm{ab}}$	1.48	1.79	1.61	0.81${}^{\mathrm{abc}}$	2.42${}^{b}$	2.34
B${}_{3}$	48.20	71.73	59.67	34.96${}^{a}$	44.32	39.25${}^{a}$	1.45	1.84	1.60	0.80${}^{\mathrm{bc}}$	3.00${}^{a}$	2.40
B${}_{4}$	47.24	69.88	57.68	33.77${}^{\mathrm{ab}}$	43.42	37.10${}^{\mathrm{ab}}$	1.40	1.73	1.63	0.87${}^{a}$	2.38${}^{b}$	2.35
Orthogonal contrast												
A	${}^{*}$	${}^{*}$	${}^{*}$	${}^{*}$	NS	${}^{*}$	${}^{*}$	NS	${}^{*}$	${}^{**}$	NS	NS
B	${}^{*}$	${}^{*}$	${}^{*}$	${}^{*}$	NS	${}^{*}$	${}^{*}$	NS	${}^{*}$	${}^{*}$	${}^{**}$	NS
A × B	NS	NS	${}^{*}$	${}^{*}$	${}^{*}$	${}^{*}$	${}^{*}$	${}^{*}$	${}^{*}$	NS	${}^{**}$	${}^{*}$

The groups A${}_{1}$, A${}_{2}$, A${}_{3}$ and A${}_{4}$ were treated by glutamine at 0, 0.4, 0.6 and 0.8 percentage of diet weight, and groups B${}_{1}$, B${}_{2}$, B${}_{3}$ and B${}_{4}$ were treated by L-carnitine at 0, 50, 75 and 100 mg/kg diet weight. ${}^{\text{a–c}}$ Mean values within a column with no common superscript differ significantly (P<0.05). ${}^{*}$ P<0.05. ${}^{**}$ P<0.01. NS: not significant.

L-carnitine had significant impact on the relative weights of thymus (P<0.05) while glutamine lacked this effect (P>0.05), and groups A${}_{1}$ had low values, but groups B${}_{1}$ or A${}_{1}$B${}_{1}$ had the lowest means. The relative weights of the spleen were strongly affected by glutamine or L-carnitine (P<0.05), and groups A${}_{1}$ or B${}_{1}$ or A${}_{1}$B${}_{1}$ had the lowest means. The influence of glutamine or L-carnitine on the relative weights of bursa of Fabricius was not obvious (P>0.05), but still groups A${}_{1}$ or B${}_{1}$ or A${}_{1}$B${}_{1}$ had the lowest values.

### 3.2 Effects of glutamine and L-carnitine on related hormones and biochemical indexes in broilers under cold stress

The plasma concentrations of CORT, T${}_{3}$ and T${}_{4}$ are shown in Table 5, the plasma concentrations of GLU, TP, BUN and ALP in Table 6, and the results of orthogonal contrast and variance analysis in Table 7. In the first week, CORT concentrations were not affected by different levels of glutamine or L-carnitine (P>0.05), and there was no interaction (P>0.05). However, in the second week the interaction appeared (P<0.05) and glutamine created obvious impact (P<0.05), and groups A${}_{1}$ or B${}_{1}$ had the highest values. The group A${}_{1}$B${}_{1}$ also had the highest plasma CORT, but in the 2 weeks the exposure time did not affect plasma CORT levels (P>0.05).

**Table 5:** The plasma corticosterone and thyroid hormone of Arbor Acres broilers under low temperature conditions supplemented by glutamine and L-carnitine.

Group	Corticosterone (ng/mL)		Triiodothyronine (pmol/L)		Thyroxine (pmol/L)	
	2 week	3 week	2 week	3 week	2 week	3 week
A${}_{1}$B${}_{1}$	17.62 ± 4.25	20.47 ± 6.21	0.84 ± 0.11	4.06 ± 1.29	9.30 ± 0.30	8.61 ± 0.70
A${}_{1}$B${}_{2}$	15.38 ± 1.16	19.57 ± 1.21	1.92 ± 0.79	5.86 ± 3.03	14.22 ± 7.06	8.93 ± 2.71
A${}_{1}$B${}_{3}$	15.05 ± 2.88	18.94 ± 1.84	1.65 ± 0.83	4.43 ± 0.75	17.02 ± 4.35	12.00 ± 3.90
A${}_{1}$B${}_{4}$	13.44 ± 0.32	13.85 ± 2.02	1.37 ± 0.45	4.20 ± 0.79	16.57 ± 0.16	9.19 ± 1.28
A${}_{2}$B${}_{1}$	16.47 ± 1.78	17.96 ± 1.58	1.91 ± 0.40	4.31 ± 0.61	9.39 ± 0.89	10.63 ± 2.38
A${}_{2}$B${}_{2}$	14.37 ± 2.39	14.36 ± 0.77	1.66 ± 0.25	4.11 ± 0.08	14.04 ± 1.17	16.23 ± 5.52
A${}_{2}$B${}_{3}$	14.26 ± 1.75	13.05 ± 2.16	1.69 ± 0.86	4.99 ± 4.94	15.49 ± 3.51	11.92 ± 4.90
A${}_{2}$B${}_{4}$	15.15 ± 3.71	13.59 ± 2.41	1.83 ± 0.33	7.82 ± 2.34	18.39 ± 5.09	9.20 ± 4.80
A${}_{3}$B${}_{1}$	15.22 ± 3.21	16.27 ± 5.03	1.64 ± 0.33	4.19 ± 1.15	11.82 ± 5.79	13.19 ± 6.07
A${}_{3}$B${}_{2}$	12.58 ± 1.39	15.41 ± 2.07	1.86 ± 0.32	7.76 ± 1.14	27.24 ± 12.88	12.24 ± 2.77
A${}_{3}$B${}_{3}$	12.61 ± 1.31	14.84 ± 1.20	1.55 ± 0.34	4.23 ± 1.34	21.72 ± 5.44	19.99 ± 2.27
A${}_{3}$B${}_{4}$	13.90 ± 1.97	16.29 ± 2.05	1.96 ± 0.22	7.18 ± 2.38	21.57 ± 5.44	10.37 ± 2.21
A${}_{4}$B${}_{1}$	13.77 ± 0.88	12.97 ± 1.69	1.28 ± 0.12	6.45 ± 2.89	10.90 ± 3.88	11.00 ± 3.62
A${}_{4}$B${}_{2}$	14.47 ± 1.97	17.60 ± 2.18	1.19 ± 0.45	7.64 ± 2.70	24.38 ± 10.08	17.04 ± 8.78
A${}_{4}$B${}_{3}$	12.39 ± 2.00	14.92 ± 2.37	2.08 ± 0.35	5.31 ± 1.61	16.40 ± 6.68	18.69 ± 7.68
A${}_{4}$B${}_{4}$	13.08 ± 1.72	12.87 ± 1.07	1.36 ± 0.33	7.94 ± 2.31	18.37 ± 7.29	10.57 ± 3.71

The groups A${}_{1}$, A${}_{2}$, A${}_{3}$ and A${}_{4}$ were treated by glutamine at 0, 0.4, 0.6 and 0.8 percentage of diet weight, and groups B${}_{1}$, B${}_{2}$, B${}_{3}$ and B${}_{4}$ were treated by L-carnitine at 0, 50, 75 and 100 mg/kg diet weight. Data are given as mean ± SEM.

**Table 6:** The plasma parameters of Arbor Acres broilers under low temperature conditions supplemented by glutamine and L-carnitine.

Group	Glucose		Total protein		Blood urea		Alkaline phosphatase	
	(mmol/L)		(g/L)		nitrogen (mmol/L)		(U/L)	
	2 week	3 week	2 week	3 week	2 week	3 week	2 week	3 week
A${}_{1}$B${}_{1}$	8.51 ± 0.98	8.80 ± 3.01	19.72 ± 2.38	22.24 ± 4.49	2.77 ± 0.35	2.28 ± 0.71	3416.00 ± 716.32	3714.67 ± 605.26
A${}_{1}$B${}_{2}$	9.72 ± 1.36	8.97 ± 1.42	29.14 ± 1.05	27.33 ± 2.11	2.25 ± 0.24	1.93 ± 0.09	3016.00 ± 656.20	2562.67 ± 868.52
A${}_{1}$B${}_{3}$	9.80 ± 0.34	11.27 ± 1.29	23.57 ± 6.66	24.53 ± 2.46	2.67 ± 0.04	1.75 ± 0.08	2728.00 ± 512.38	2381.33 ± 691.72
A${}_{1}$B${}_{4}$	9.82 ± 1.27	11.82 ± 2.19	26.40 ± 4.42	29.54 ± 4.71	2.30 ± 0.14	1.87 ± 0.29	3270.33 ± 313.15	2912.00 ± 622.31
A${}_{2}$B${}_{1}$	9.06 ± 0.88	10.26 ± 0.89	20.35 ± 5.98	25.31 ± 6.15	2.70 ± 0.77	1.89 ± 0.56	3132.67 ± 631.80	2760.00 ± 505.68
A${}_{2}$B${}_{2}$	12.73 ± 2.69	12.29 ± 3.43	22.09 ± 1.58	33.16 ± 1.06	2.43 ± 0.05	1.70 ± 0.26	2841.00 ± 666.77	2588.00 ± 633.94
A${}_{2}$B${}_{3}$	11.47 ± 1.64	10.29 ± 2.92	23.51 ± 1.59	37.70 ± 4.50	2.50 ± 0.20	1.83 ± 0.22	2840.00 ± 778.96	2448.00 ± 605.28
A${}_{2}$B${}_{4}$	12.93 ± 2.08	12.04 ± 2.01	25.04 ± 1.14	32.24 ± 4.49	2.51 ± 0.26	1.68 ± 0.13	2505.00 ± 692.96	2276.67 ± 677.38
A${}_{3}$B${}_{1}$	9.40 ± 0.53	10.90 ± 2.83	24.44 ± 4.11	26.23 ± 7.77	2.62 ± 0.42	2.08 ± 0.13	2418.67 ± 392.60	2548.00 ± 620.62
A${}_{3}$B${}_{2}$	11.06 ± 0.35	13.63 ± 2.65	25.33 ± 1.81	37.53 ± 2.67	2.30 ± 0.18	2.11 ± 0.26	2804.00 ± 541.42	2312.00 ± 598.31
A${}_{3}$B${}_{3}$	14.02 ± 5.02	9.56 ± 1.59	23.40 ± 3.32	31.97 ± 6.97	2.42 ± 0.27	1.62 ± 0.18	2925.00 ± 218.36	2672.00 ± 893.78
A${}_{3}$B${}_{4}$	15.05 ± 3.22	12.15 ± 1.79	27.88 ± 9.26	35.62 ± 7.68	2.46 ± 0.04	1.69 ± 0.09	2680.00 ± 324.12	3016.00 ± 844.51
A${}_{4}$B${}_{1}$	9.81 ± 1.77	12.82 ± 4.70	26.24 ± 6.22	30.00 ± 10.84	2.32 ± 0.16	1.85 ± 0.49	3165.33 ± 750.82	2330.67 ± 501.16
A${}_{4}$B${}_{2}$	13.00 ± 3.60	11.07 ± 2.13	22.86 ± 4.01	35.56 ± 7.48	2.07 ± 0.07	1.82 ± 0.16	2878.33 ± 735.54	2912.00 ± 912.28
A${}_{4}$B${}_{3}$	11.86 ± 3.42	13.86 ± 3.27	32.95 ± 4.43	41.30 ± 3.89	2.34 ± 0.05	1.93 ± 0.32	3131.50 ± 707.81	2272.00 ± 784.66
A${}_{4}$B${}_{4}$	14.42 ± 3.28	8.88 ± 1.08	30.06 ± 3.32	29.42 ± 5.47	2.28 ± 0.18	1.79 ± 0.13	2337.33 ± 639.82	2880.00 ± 764.32

The groups A${}_{1}$, A${}_{2}$, A${}_{3}$ and A${}_{4}$ were treated by glutamine at 0, 0.4, 0.6 and 0.8 percentage of diet weight, and groups B${}_{1}$, B${}_{2}$, B${}_{3}$ and B${}_{4}$ were treated by L-carnitine at 0, 50, 75 and 100 mg/kg diet weight. Data are given as mean ± SEM.

**Table 7:** The effects of supplemented glutamine and L-carnitine on corticosterone, thyroid hormone and other parameters of Arbor Acres broilers under low temperature conditions.

Group	Corticosterone		Triiodothyronine		Thyroxine		Glucose		Total protein		Blood urea		Alkaline	
	(ng/mL)		(pmol/L)		(pmol/L)		(mmol/L)		(g/L)		nitrogen		phosphatase	
											(mmol/L)		(U/L)	
	2	3	2	3	2	3	2	3	2	3	2	3	2	3
	week	week	week	week	week	week	week	week	week	week	week	week	week	week
Glutamine														
A${}_{1}$	15.29	18.21${}^{a}$	1.21${}^{b}$	5.99${}^{b}$	10.66${}^{c}$	8.93${}^{b}$	9.45${}^{b}$	11.04	20.72${}^{c}$	26.46${}^{b}$	2.50	1.88	2991.00	3132.67${}^{a}$
A${}_{2}$	15.61	15.84${}^{b}$	1.92${}^{\mathrm{ab}}$	5.31${}^{b}$	14.55${}^{b}$	13.83${}^{\mathrm{ab}}$	11.63${}^{a}$	10.99	26.61${}^{\mathrm{ab}}$	29.11${}^{\mathrm{ab}}$	2.45	1.87	2829.67	2567.67${}^{b}$
A${}_{3}$	14.59	15.70${}^{\mathrm{ab}}$	2.10${}^{\mathrm{ab}}$	6.32${}^{\mathrm{ab}}$	19.87${}^{a}$	14.33${}^{a}$	12.49${}^{a}$	11.09	23.62${}^{\mathrm{bc}}$	31.45${}^{a}$	2.43	1.86	2828.50	2537.00${}^{b}$
A${}_{4}$	13.26	13.34${}^{b}$	2.69${}^{a}$	9.05${}^{a}$	16.88${}^{\mathrm{ab}}$	10.86${}^{\mathrm{ab}}$	12.08${}^{a}$	11.49	27.29${}^{a}$	32.52${}^{a}$	2.39	1.84	2803.13	2108.67${}^{b}$
L-carnitine														
B${}_{1}$	15.11	16.77	1.61	5.58	13.23	10.57	9.50${}^{b}$	9.99${}^{b}$	22.36${}^{b}$	23.92	2.41	1.93	2807.00	2897.67${}^{b}$
B${}_{2}$	14.24	15.60	2.00	7.57	16.00	11.57	11.32${}^{a}$	11.19${}^{\mathrm{ab}}$	25.50${}^{a}$	31.94	2.39	1.90	3001.42	2792.00${}^{b}$
B${}_{3}$	14.00	15.42	2.16	5. 38	16.46	13.61	11.79${}^{a}$	12.47${}^{a}$	25.53${}^{a}$	33.58	2.57	1.76	2979.29	2378.67${}^{\mathrm{ab}}$
B${}_{4}$	14.41	15.31	2.15	7.69	16.27	12.20	13.06${}^{a}$	10.96${}^{\mathrm{ab}}$	24.86${}^{a}$	30.09	2.43	1.86	2659.58	2277.67${}^{a}$
Orthogonal contrast														
A	NS	${}^{**}$	NS	NS	${}^{*}$	${}^{*}$	${}^{*}$	${}^{*}$	${}^{**}$	${}^{**}$	NS	${}^{*}$	NS	${}^{*}$
B	NS	NS	NS	NS	NS	${}^{*}$	${}^{*}$	${}^{*}$	${}^{*}$	${}^{*}$	NS	NS	NS	${}^{*}$
A × B	NS	${}^{*}$	NS	NS	${}^{**}$	${}^{**}$	NS	${}^{*}$	NS	${}^{*}$	NS	${}^{*}$	NS	${}^{*}$

The groups A${}_{1}$, A${}_{2}$, A${}_{3}$ and A${}_{4}$ were treated by glutamine at 0, 0.4, 0.6 and 0.8 percentage of diet weight, and groups B${}_{1}$, B${}_{2}$, B${}_{3}$ and B${}_{4}$ were treated by L-carnitine at 0, 50, 75 and 100 mg/kg diet weight. ${}^{\text{a–c}}$ Mean values within a column with no common superscript differ significantly (P<0.05). ${}^{*}$ P<0.05. ${}^{**}$ P<0.01. NS: not significant.

There was no influence of glutamine or L-carnitine or interaction on the plasma T${}_{3}$ (P>0.05), and group A${}_{1}$B${}_{1}$ had the least values. Groups A${}_{1}$ or B${}_{1}$ had the lowest T${}_{3}$ levels in the first week while having relatively low levels in the second week. On the other hand, the two additives had obviously influenced the plasma concentrations of T${}_{4}$ (P<0.05) except the supplementation of L-carnitine in the first week (P>0.05), and there was the interaction (P<0.05). Groups A${}_{1}$ or B${}_{1}$ have the significantly lowest T${}_{4}$ (P<0.05), while the values of groups A${}_{3}$ or B${}_{3}$ are the highest. Moreover, the change of T${}_{3}$ or T${}_{4}$ had the time-dependent effect (P<0.05), and from the first week to second week T${}_{3}$ grew obviously (P<0.05), while T${}_{4}$ declined significantly (P<0.05).

The plasma concentrations of GLU, TP, BUN and ALP had been altered by the dietary supplementation. The addition of glutamine or L-carnitine apparently altered plasma GLU concentrations (P<0.05), though only in the second week there was obvious interaction (P<0.05). In the first week, groups A${}_{1}$ or B${}_{1}$ obviously had the smallest values (P<0.05), while in the second week, the means of groups A${}_{1}$ were not the least but not different from the others, and the levels of groups B${}_{1}$ still were the lowest. Moreover, the other groups held relatively stable and higher plasma GLU, and the chickens in A${}_{1}$B${}_{1}$ always had the lowest GLU concentrations. The change profile of TP was largely identical to that of GLU, but the glutamine had a more obvious effect (P<0.05), and all TP contents of groups A${}_{1}$ or B${}_{1}$ or A${}_{1}$B${}_{1}$ were the lowest. Contrarily, only in the second week, dietary glutamine had a great impact on the plasma BUN levels (P<0.05), but the means of groups A${}_{1}$ were not significantly different from others (P>0.05). Nonetheless, the BUN means of all groups decreased obviously (P<0.05) and the chickens in group A${}_{1}$B${}_{1}$ had the highest BUN levels. The alteration of ALP activity caused by the added glutamine or L-carnitine and the interaction became apparent only in the second week (P<0.05), and groups A${}_{1}$ or B${}_{1}$ had the biggest means. With the exposure, groups A${}_{1}$ or B${}_{1}$ had increased ALP activity, whereas those of the left groups declined. Overall, group A${}_{1}$B${}_{1}$ always had the highest level.

### 3.3 Effects of glutamine and L-carnitine on antioxidant capacity of broilers under cold stress

The data of plasma SOD, GHS-PX, T-AOC and MDA are presented in the Table 8, and the results of orthogonal contrast and variance analysis can be found in Table 9. The activity of SOD and GHS-XP was not affected by the added L-carnitine (P>0.05), but it changed obviously because of the supplemented glutamine (P<0.05) and in the second week by the obvious interaction (P<0.05). In the studied 2 weeks, SOD of groups A${}_{1}$ or B${}_{1}$ held the relatively stable level, whereas those in the other groups grew significantly (P<0.05). The added glutamine had the positive dose-dependent impact on the GHS-PX activity (P<0.05), and groups B${}_{1}$ had the lowest levels, though the L-carnitine had no obvious effect (P>0.05). The chickens in group A${}_{1}$B${}_{1}$ had the lowest activity of SOD and GSH-PX. With regard to T-AOC, both glutamine and L-carnitine yielded the obvious effect (P<0.05), though only in the second week the interaction became apparent (P<0.05). The means of all groups increased with the experimental period, and groups A${}_{4}$ or B${}_{4}$ had the highest values and gained the most, whereas groups A${}_{1}$ or B${}_{1}$ had the lowest means and grew the least. Moreover, group A${}_{1}$B${}_{1}$ had the lowest T-AOC. L-carnitine had obvious influence on plasma MDA (P<0.05) and groups B${}_{1}$ had the highest levels. In the second week, glutamine had the significant effect (P<0.05), and groups A${}_{1}$ had the highest levels but no significant difference from others (P>0.05). Both additives did not exert the time-dependent effect on the change of plasma MDA (P>0.05), but the interaction always existed (P<0.05) and group A${}_{1}$B${}_{1}$ had the highest levels.

**Table 8:** The antioxidant capacity of Arbor Acres broilers under low temperature conditions supplemented by glutamine and L-carnitine.

Group	Superoxide dismutase		Glutathione peroxidase		Total antioxidant capacity		Malondialdehyde	
	(U/mL)		(U/mL)		(antioxidant unit/mL)		(nmol/mL)	
	2 week	3 week	2 week	3 week	2 week	3 week	2 week	3 week
A${}_{1}$B${}_{1}$	59.62 ± 6.99	97.94 ± 1.59	321.58 ± 6.14	324.68 ± 7.78	5.80 ± 1.11	12.98 ± 1.67	5.39 ± 0.12	5.83 ± 0.72
A${}_{1}$B${}_{2}$	74.56 ± 1.30	150.31 ± 5.11	395.52 ± 9.62	386.63 ± 5.87	12.81 ± 9.91	32.81 ± 9.47	3.05 ± 0.92	3.11 ± 0.35
A${}_{1}$B${}_{3}$	97.97 ± 1.29	140.83 ± 1.42	324.70 ± 8.35	326.84 ± 3.52	10.31 ± 1.27	26.98 ± 3.89	1.97 ± 0.31	3.90 ± 0.76
A${}_{1}$B${}_{4}$	72.99 ± 1.49	113.05 ± 0.89	332.01 ± 7.21	335.48 ± 6.50	12.24 ± 1.40	35.07 ± 2.18	2.62 ± 0.99	3.78 ± 0.86
A${}_{2}$B${}_{1}$	127.73 ± 2.62	141.86 ± 3.54	438.03 ± 7.33	428.99 ± 6.14	12.41 ± 2.76	16.92 ± 3.12	2.08 ± 0.16	4.33 ± 1.45
A${}_{2}$B${}_{2}$	103.31 ± 4.04	129.19 ± 3.92	338.58 ± 1.66	385.25 ± 6.60	9.87 ± 1.40	29.51 ± 2.18	2.31 ± 0.56	2.11 ± 0.59
A${}_{2}$B${}_{3}$	110.65 ± 5.28	148.97 ± 5.81	433.72 ± 3.80	421.36 ± 2.48	16.11 ± 2.93	26.79 ± 3.63	3.10 ± 0.65	2.36 ± 0.54
A${}_{2}$B${}_{4}$	122.66 ± 1.35	127.71 ± 4.36	440.28 ± 5.13	336.37 ± 3.70	16.27 ± 4.05	28.27 ± 2.21	2.41 ± 0.11	2.48 ± 0.54
A${}_{3}$B${}_{1}$	118.69 ± 2.16	128.43 ± 0.57	362.41 ± 5.47	346.90 ± 8.36	5.89 ± 1.15	21.43 ± 3.40	1.49 ± 0.85	2.95 ± 0.48
A${}_{3}$B${}_{2}$	117.50 ± 3.96	130.46 ± 6.87	394.14 ± 3.34	402.66 ± 7.04	8.80 ± 1.13	22.24 ± 4.66	3.59 ± 0.19	3.31 ± 0.96
A${}_{3}$B${}_{3}$	112.43 ± 5.96	156.71 ± 5.24	347.96 ± 5.68	441.15 ± 6.31	9.67 ± 6.84	31.28 ± 3.43	1.46 ± 0.11	2.82 ± 0.49
A${}_{3}$B${}_{4}$	93.17 ± 1.19	159.01 ± 1.72	364.55 ± 3.78	371.15 ± 4.32	10.51 ± 0.01	38.05 ± 2.43	1.62 ± 0.11	2.66 ± 0.56
A${}_{4}$B${}_{1}$	140.26 ± 1.95	156.07 ± 1.58	386.53 ± 8.73	344.97 ± 7.79	15.47 ± 4.19	28.36 ± 5.21	2.31 ± 0.97	2.50 ± 0.09
A${}_{4}$B${}_{2}$	130.71 ± 2.55	142.38 ± 2.75	459.05 ± 2.63	352.57 ± 4.11	13.78 ± 3.75	32.24 ± 3.11	2.26 ± 0.69	3.52 ± 0.41
A${}_{4}$B${}_{3}$	168.05 ± 3.99	169.02 ± 1.99	382.56 ± 7.70	436.21 ± 6.96	17.27 ± 2.78	56.07 ± 8.19	4.00 ± 0.21	4.56 ± 0.20
A${}_{4}$B${}_{4}$	154.59 ± 3.82	178.51 ± 2.40	373.74 ± 6.33	353.65 ± 8.11	18.69 ± 3.50	35.79 ± 1.70	1.80 ± 0.13	2.40 ± 0.42

The groups A${}_{1}$, A${}_{2}$, A${}_{3}$ and A${}_{4}$ were treated by glutamine at 0, 0.4, 0.6 and 0.8 percentage of diet weight, and groups B${}_{1}$, B${}_{2}$, B${}_{3}$ and B${}_{4}$ were treated by L-carnitine at 0, 50, 75 and 100 mg/kg diet weight. Data are given as mean ± SEM.

**Table 9:** The effects of supplemented glutamine and L-carnitine on antioxidant capacity of Arbor Acres broilers under low temperature conditions.

Group	Superoxide		Glutathione		Total antioxidant		Malondialdehyde	
	dismutase		peroxidase		capacity (antioxidant		(nmol/mL)	
	(U/mL)		(U/mL)		unit/mL)			
	2 week	3 week	2 week	3 week	2 week	3 week	2 week	3 week
Glutamine								
A${}_{1}$	128.98${}^{a}$	125.53${}^{b}$	324.77${}^{b}$	321.67${}^{b}$	12.91${}^{b}$	11.96${}^{b}$	3.01	2.96
A${}_{2}$	95.96${}^{b}$	134.43${}^{b}$	375.97${}^{\mathrm{ab}}$	344.24${}^{\;\mathrm{ab}}$	13.70${}^{b}$	15.75${}^{b}$	2.72	2.77
A${}_{3}$	111.64${}^{\mathrm{ab}}$	135.15${}^{\mathrm{ab}}$	416.09${}^{\mathrm{ab}}$	413.60${}^{\mathrm{ab}}$	6.44${}^{c}$	18.45${}^{b}$	2.23	2.68
A${}_{4}$	130.05${}^{a}$	149.08${}^{a}$	423.40${}^{a}$	418.55${}^{\;a}$	16.05${}^{\mathrm{ab}}$	47.25${}^{a}$	3.09	2.75
L-carnitine								
B${}_{1}$	124.33	122.57	326.44	325.27	12.49${}^{\mathrm{ab}}$	23.35${}^{b}$	3.62${}^{a}$	3.15${}^{a}$
B${}_{2}$	109.44	135.17	413.41	389.38	12.75${}^{\mathrm{ab}}$	22.14${}^{b}$	2.62${}^{\mathrm{ab}}$	2.82${}^{\mathrm{ab}}$
B${}_{3}$	124.83	148.88	397.23	408.24	9.65${}^{b}$	23.04${}^{b}$	1.83${}^{b}$	2.34${}^{b}$
B${}_{4}$	108.05	137.57	403.14	398.02	14.22${}^{a}$	38.40${}^{a}$	3.00${}^{\mathrm{ab}}$	2.83${}^{\mathrm{ab}}$
Orthogonal contrast								
A	${}^{*}$	${}^{*}$	${}^{*}$	${}^{*}$	${}^{**}$	${}^{**}$	NS	${}^{*}$
B	NS	NS	NS	NS	${}^{*}$	${}^{**}$	${}^{*}$	${}^{*}$
A × B	${}^{**}$	${}^{**}$	NS	${}^{*}$	NS	${}^{**}$	${}^{*}$	${}^{**}$

The groups A${}_{1}$, A${}_{2}$, A${}_{3}$ and A${}_{4}$ were treated by glutamine at 0, 0.4, 0.6 and 0.8 percentage of diet weight, and groups B${}_{1}$, B${}_{2}$, B${}_{3}$ and B${}_{4}$ were treated by L-carnitine at 0, 50, 75 and 100 mg/kg diet weight. ${}^{\text{a–c}}$ Mean values within a column with no common superscript differ significantly (P<0.05). ${}^{*}$ P<0.05. ${}^{**}$ P<0.01. NS: not significant.

## 4 Discussion

The inconspicuous effect of glutamine and L-carnitine on plasma CORT in the first week and the strong impact of glutamine and obvious interaction in the second week may indicate that only in the second week the ability of glutamine and L-carnitine to offset the increasing of plasma CORT becomes apparent. Since elevated levels of plasma CORT are a classic indicator of stress (Quinteiro-Filho et al., 2012; Honda et al., 2015), the highest levels of plasma CORT in groups A${}_{1}$ or B${}_{1}$ or A${}_{1}$B${}_{1}$ imply that the dietary glutamine and L-carnitine might have the ability to reduce the plasma CORT concentrations and thereby could ameliorate the stress responses or improve the resistance of the broilers to cold stress. Glutamine or L-carnitine has been used as effectively dietary fortification to resist various stressful situations (Izgüt-Uysal et al., 2007; Kelek et al., 2019).

The groups A${}_{1}$ or B${}_{1}$ or A${}_{1}$B${}_{1}$ have the highest DFI, relatively lower DWG and the highest F / G, and the supplementary glutamine and L-carnitine have the obvious interaction, though there is significant difference among groups F / G only in the first week. It can be concluded that the dietary supplementation elicited the positive responses of growth performance and feed conversion in the broilers during the 2-week exposure to low temperature, and the studies about many species under various conditions have the same report (Yi et al., 2005; Bartell and Batal, 2007). Obviously, the groups A${}_{1}$ or B${}_{1}$ or A${}_{1}$B${}_{1}$ have poor growth performance and feed efficiency, mainly because they have spent more energy for thermogenesis to resist the cold stress rather than for growth, and thus their energy metabolism has been damaged (Hangalapura et al., 2003; King and Swanson, 2013).

One possible mechanism to maintain the normal energy consumption in the additive-administrated groups is that the addition of glutamine or L-carnitine might moderate the stress-induced rising of plasma CORT and improve the impaired energy balance of broilers exposed to cold, which might be caused by the higher plasma CORT (Yuan et al., 2008), and the obvious interaction of the two additives strengthens the effect. The high levels of stress-induced CORT may stimulate the higher energy to spend for various stress responses and hurt the energy efficiency (Jiang et al., 2008; Yuan et al., 2008; Babacanoğlu et al., 2013), which may result in the redistribution of energy and consequently damage the growth of many species (Ralph et al., 2015).

Another involved mechanism is the alteration of thyroid hormone that would induce the better energy metabolism (Chatzitomaris et al., 2017; Nordio, 2017). Glutamine- or L-carnitine-supplemented groups have higher T${}_{3}$ and T${}_{4}$ than groups A${}_{1}$ or B${}_{1}$, and therefore the chickens in the latter groups might hold poorer metabolic conditions and hence should have worse growth performance. The obvious variance of thyroid hormone may be negatively correlated to the CORT concentrations as some research has found (Angelier et al., 2016). The plasma CORT in groups A${}_{1}$ or B${}_{1}$ or A${}_{1}$B${}_{1}$ is the highest, and consequently T${}_{3}$ and T${}_{4}$ levels are lowest. Furthermore, the reduction of T${}_{4}$ and increasing of T${}_{3}$ with the exposure time in all groups means the augmented transformation of inactive T${}_{4}$ to active T${}_{3}$ probably meets the strong metabolic need of the 1-day-old chickens for growth or stress resistance in the experimental 2 weeks.

Glutamine not only is an important precursor for the synthesis of amino acids, nucleotides, nucleic acids, amino sugars, proteins and many other biologically important molecules, but also has been demonstrated to be the principal metabolic fuel for rapidly proliferating cells (Yi et al., 2005; Roth, 2008; Wellen et al., 2010). Furthermore, diets supplemented with L-carnitine would enhance the oxidation of long-chain fatty acids to generate ATP, improve energy efficiency (Rehman et al., 2017), consequently perhaps promote the protein-sparing action of fat (Li et al., 2017), reduce the heat production in exercising pigeons (Celik and Oztürkcan, 2003), and has been reported to have a protective effect on gastric mucosal barrier in rats exposed to cold-restraint stress (Izgüt-Uysal et al., 2007). Thus, there are still maybe other approaches for glutamine and L-carnitine to improve the metabolism for the young broilers at rapid development.

The relative weights of the spleen, thymus and bursa of Fabricius in the groups A${}_{1}$ or B${}_{1}$ are low, and group A${}_{1}$B${}_{1}$ has the lowest values, which implies that immunodepression happened. These results confirm that the immunological organs are sensitive to CORT (Mehaisen et al., 2017; Yaribeygi et al., 2017), for the highest plasma CORT levels were observed in the groups A${}_{1}$ or B${}_{1}$ or A${}_{1}$B${}_{1}$. Broilers are a kind of homothermal animal that have a high energy requirement for thermoregulation under low temperature, which is probably a constraint to immune function (Hangalapura et al., 2003), and hence energy-demanding immune responses in the control group A${}_{1}$B${}_{1}$ or groups A${}_{1}$ or B${}_{1}$ may be suppressed. Therefore, the supplementation of glutamine and L-carnitine has mitigated the induced immunodepression by moderating augmentation of plasma CORT and keeping the energy balance, which will consequently improve the immunity of broilers raised under low temperature.

The glutamine or L-carnitine supplemented groups keep relatively stable plasma GLU in the experiment period since the CORT concentration had no obvious fluctuation, and then the CORT-induced glycogenolysis or gluconeogenesis and hyperglycemia will not occur apparently (Zhao et al., 2009). Moreover, glutamine or L-carnitine addition is able to arouse the increasing of plasma glucose because glutamine is one of the most effective substrates for gluconeogenesis and L-carnitine participates in biological regulation of gluconeogenesis involving the reduced oxidation of gluconeogenic precursors and the favored aerobic metabolism (Celik and Oztürkcan, 2003; Roth, 2008; Ringseis et al., 2013). However, it is the significantly heightened CORT in the groups A${}_{1}$ that incited the increase of GLU in the second week.

Plasma TP levels are regarded as an index of protein nutritional status. Thereby, the dietary supplemented groups obviously have better protein nutritional conditions, and group A${}_{1}$B${}_{1}$ has the poorest status. The addition of carnitine to animal feed can promote the oxidation of fat, which provides the most cost-effective energy and has the potential to enhance sparing of the protein (Rehman et al., 2017). In humans, L-carnitine supplement was reported to improve the serum total protein concentrations in patients in maintenance dialysis by sparing nitrogen because fewer amino acids are required for synthesis of L-carnitine (Katalinic et al., 2018). In addition, glutamine is 1 of the 20 proteinogenic amino acids accounting for 5 %–6 % of bound amino acids, and it is also the most abundant amino acid in blood and the free amino acid pool (Roth, 2008). Glutamine has been reported to improve nitrogen balance in a catabolic state (Buijs et al., 2013). However, there is no conspicuously different BUN between all groups, which indicates that CORT-induced protein catabolism does not happen because of the short stress period or the dietary addition of glutamine or L-carnitine implies no impact on protein catabolism, and the negative time-dependent effect of plasma BUN suggests the dropping protein catabolism. Higher protein nutritional situations and declined protein catabolism in the supplemented groups might result in faster growth of these young chickens. It has been reported that glutamine may also act as a signal or regulator of metabolic demands to increase protein synthesis and decrease protein degradation in skeletal muscle of young broilers (Xi et al., 2011; Lambertucci et al., 2012). Conversely, group A${}_{1}$B${}_{1}$ without glutamine or L-carnitine supplementation owns the lowest TP and highest BUN and then poorer growth performance.

The higher ALP activity of groups A${}_{1}$ or B${}_{1}$ or A${}_{1}$B${}_{1}$ in the second week may illustrate the bone metabolism, and thus be induced by bone abnormality, which is one of a series of responses to the higher CORT levels of these broilers because the high glucocorticoid may seriously hurt the physiological conditions of the skeletal system (Beier et al., 2017). Although ALP activity appears to be associated with bone activity, the intestinal isoenzyme makes the largest contribution to plasma ALP activity, and the injury of intestinal mucosa may cause the increasing of plasma ALP (Viveros et al., 2002). Glutamine might play the important role in maintaining the integrity of the intestinal mucosa as the fuel of the cells of the intestinal mucosa and the gut-associated lymphoid tissue (Bartell and Batal, 2007; Izgüt-Uysal et al., 2007). Therefore, the negative time-dependent effect of ALP activity in the glutamine or L-carnitine supplemented groups may be the response to not rising CORT levels or the protective function of the additives to the intestinal mucosa.

The supplementation of glutamine or L-carnitine has ameliorated the oxidative stress and improved the antioxidant capability, and their significant interaction, especially in the second week, has fortified the results. The corticosterone-induced or stress-induced oxidative stress, as many studies have found, should be not serious in the supplemented groups, for the CORT level was decreased by the additives (Lin et al., 2004). Moreover, glutamine is a precursor of glutathione, and then its supplementation can be used to maintain high levels of glutathione (by the altered activity of GHS-PX) and to avoid oxidative stress damage (Roth, 2008). In this experiment, the addition of glutamine has obviously improved plasma SOD and GHS-PX activity and nonenzymatic antioxidants (T-AOC) and decreased plasma lipid peroxidation (estimated by MDA levels), and the variation of GHS-PX activity is both dose- and time-dependent, especially in the supplemented groups.

The apparently unmodified activity of plasma SOD and GHS-PX by the added L-carnitine is against what was observed in mammals and might be caused by the short period of the exposure and the less reliability on the energy production by lipids (Buijs et al., 2013; Katalinic et al., 2018). Nonetheless, since L-carnitine would facilitate the oxidation of long-chain fatty acids and aerobic metabolism, L-carnitine obviously reduced the plasma concentrations of MDA and hence mollified the lipid peroxidation, which would be supported by the trials of humans and rats (Banihani et al., 2012; Jia et al., 2014; Kazemi Fard et al., 2015). Moreover, the vitamin C and methionine sparing activity of L-carnitine can maintain the levels of antioxidants, and therefore T-AOC grew with the addition of L-carnitine (Roth, 2008). The inconspicuous changes of plasma MDA concentrations with time in all groups indicate the stable lipid peroxidation, which might be due to the simultaneous rise in T-AOC, suggesting the increased antioxidant capacity with age.

## 5 Conclusion

The supplemented diet of glutamine and L-carnitine can improve the growth performance and nutritional status and ameliorate the stress response and oxidative stress of the young broilers exposed to low ambient temperature. Their interaction, especially significant in the second week, has amplified the results. The orthogonal contrast analysis shows that the group A${}_{3}$B${}_{4}$ had the highest feed efficiency, and then the broilers that were raised at the low ambient temperature in this experiment had better be feed by the supplement of glutamine at 0.6 percentage of diet weight and L-carnitine at 100 mg/kg diet weight.

## Data Availability

The data are available from the corresponding author upon request.
